# A parallel and incremental algorithm for efficient unique signature discovery on DNA databases

**DOI:** 10.1186/1471-2105-11-132

**Published:** 2010-03-16

**Authors:** Hsiao Ping Lee, Tzu-Fang Sheu, Chuan Yi Tang

**Affiliations:** 1 Department of Applied Information Sciences, Chung Shan Medical University, Taichung, 40201 Taiwan, ROC; 2 Department of Medical Research, Chung Shan Medical University Hospital, Taichung, 40201 Taiwan, ROC; 3 Department of Computer Science, National Tsing Hua University, Hsinchu, Taiwan, ROC; 4 Department of Computer Science and Communication Engineering, Providence University, Taichung, 43301 Taiwan, ROC

## Abstract

**Background:**

DNA signatures are distinct short nucleotide sequences that provide valuable information that is used for various purposes, such as the design of Polymerase Chain Reaction primers and microarray experiments. Biologists usually use a discovery algorithm to find unique signatures from DNA databases, and then apply the signatures to microarray experiments. Such discovery algorithms require to set some input factors, such as signature length *l *and mismatch tolerance *d*, which affect the discovery results. However, suggestions about how to select proper factor values are rare, especially when an unfamiliar DNA database is used. In most cases, biologists typically select factor values based on experience, or even by guessing. If the discovered result is unsatisfactory, biologists change the input factors of the algorithm to obtain a new result. This process is repeated until a proper result is obtained. Implicit signatures under the discovery condition (*l*, *d*) are defined as the signatures of length ≤ *l *with mismatch tolerance ≥ *d*. A discovery algorithm that could discover all implicit signatures, such that those that meet the requirements concerning the results, would be more helpful than one that depends on trial and error. However, existing discovery algorithms do not address the need to discover all implicit signatures.

**Results:**

This work proposes two discovery algorithms - the consecutive multiple discovery (CMD) algorithm and the parallel and incremental signature discovery (PISD) algorithm. The PISD algorithm is designed for efficiently discovering signatures under a certain discovery condition. The algorithm finds new results by using previously discovered results as candidates, rather than by using the whole database. The PISD algorithm further increases discovery efficiency by applying parallel computing. The CMD algorithm is designed to discover implicit signatures efficiently. It uses the PISD algorithm as a kernel routine to discover implicit signatures efficiently under every feasible discovery condition.

**Conclusions:**

The proposed algorithms discover implicit signatures efficiently. The presented CMD algorithm has up to 97% less execution time than typical sequential discovery algorithms in the discovery of implicit signatures in experiments, when eight processing cores are used.

## Background

Mutations introduce variations and divergence into DNA sequences within and among species. Differences among DNA sequences are extensively used to identify species [1-4]. For example, specific oligonucleotides have already been used in the Polymerase Chain Reaction (PCR) method to identify 14 human pathogenic yeast species [5]. A unique DNA signature is a sequence that occurs in a DNA database only once, and has some minimum mutation distance from all other sequences in the database. Unique signature discovery [6] is the finding of unique signatures in a set of DNA sequences. They are accelerating various areas of research, including the map-based cloning of genes that control traits, comparative genome analysis, protein identification, and the development of various methods that depend on gene-specific oligonucleotides, such as the DNA microarray technology.

The methods of signature discovery have been widely studied, and many related tools and applications have been developed [1,6-16]. For example, [14] integrates multiple bioinformatics algorithms to determine horizontally transferred, pathotype-specific signature genes as targets for specific, high-throughput molecular diagnostic applications and reverse vaccinology screens; insignia [15] is a web application for rapidly identifying unique DNA signatures, and hybseek [16] is a web service for efficiently designing both pathogen-specific and compatible primer pairs for DNA-based diagnostic multi-analyte assays.

The algorithm of Zheng et al. [17] and IMUS [18] are two hamming-distance-based unique signature discovery algorithms. These two algorithms deal with DNA databases. Let *l *and *d *be two positive integers, where *d *≤ *l*. An *l*-pattern is a string of *l *characters in the alphabet set {A, C, G, T}. A pattern *P *is (*l*, *d*)-mismatched to a pattern *Q *if the length of *P *and *Q *is *l *and the hamming distance, which is the number of mismatches, between *P *and *Q *does not exceed *d*. An *l*-pattern *P *is referred to as a unique signature with mismatch tolerance *d *if and only if no other pattern *Q *exists in the given DNA database such that *P *and *Q *are (*l*, *d*)-mismatched. Zheng's algorithm and the IMUS algorithm are designed for efficiently discovering the unique signatures under the discovery conditions of signature length *l *and mismatch tolerance *d*.

Zheng's algorithm, called the UO algorithm hereafter, is based on the observation that if two patterns, *P *and *Q*, are (*l*, *d*)-mismatched, then at least one of the partitions of *λ P *is (*l*/*λ*, 1)-mismatched to the corresponding part in *Q*, where *λ *= ⌊*d*/2⌋ + 1 and all partitions have equal length. The UO algorithm is a two-phase algorithm. In the first phase, the algorithm divides DNA sequences into patterns of length *l*/*λ*. An index system is built based on the *l*/*λ*-patterns as index keys, in which *l*-patterns that contain the same index key are gathered in a single index entry. Assume that *K*_*P *_is an index key, and *K*_*Q *_is one of the keys that are (*l*/*λ*, 1)-mismatched to *K*_*P*_. In the second phase, the UO algorithm performs complete string comparisons on the *l*-patterns in the entries *K*_*Q *_and *K*_*P *_to check whether they are (*l*, *d*)-mismatched. The unique signatures emerge after all of the duplicated patterns have been pruned.

The IMUS algorithm improves upon the UO algorithm. The IMUS algorithm is based on the observation that if two patterns *P *and *Q *are (*l*, *d*)-mismatched, then at least one of the two halves of *P *is (*l*/2, ⌊*d*/2⌋)-mismatched to the corresponding part of *Q*. In the processing-kernel level, the UO and IMUS algorithms are similar. The main difference between them is the number of partitions in an *l*-pattern. The IMUS algorithm divides an *l*-pattern into two partitions, whereas the UO algorithm divides a pattern into ⌊*d*/2⌋ + 1 partitions. Since the mismatch tolerance *d *is small (usually *d <*6) in most discoveries of short signatures (of length *l *≤ 40), the IMUS algorithm reduces the number of partitions in an *l*-pattern to decrease the number of required string comparisons, and thus increases the discovery efficiency. A consequence is that more memory is required to store the index that is used in the IMUS algorithm. An additional frequency filter, which represents an enhanced usage of the frequency distance, defined in [19], is used in the IMUS algorithm as a pre-filter to prevent unnecessary comparisons between dissimilar patterns. However, most signature discovery algorithms have the problem that we do not know how to select proper factor values, such as the proper (*l*, *d*) values in the UO or IMUS algorithm, because the proper discovery result is defined on a case-by-case basis. In most cases, factor values are selected based on domain knowledge or experience or even by guessing. The factor settings are then used in the discovery algorithm to discover signatures. If the result is unacceptable, then the factor values are changed to get other results. The process is repeated until satisfactory results are found. This situation often arises when an unfamiliar DNA database is being used. A method that can efficiently find all of the signatures that satisfy feasible discovery conditions, instead of repeated trial and error, enabling users to select the proper signatures, is needed. In other words, when the discovery condition is given in terms of signature length *l *and mismatch tolerance *d*, a discovery algorithm can be use to discover not only the signatures with exact (*l*, *d*) but also all signatures that meet stricter discovery conditions - with a length smaller than *l *or a mismatch tolerance larger than *d*. Then, the signatures that meet our requirements can be selected directly from the results. The signatures of length ≤ *l *and mismatch tolerance ≥ *d *are called the implicit signatures under the discovery condition (*l*, *d*). Providing researchers with all implicit signatures without manually changing the factor values would be helpful. One challenge is how to discover efficiently all implicit signatures from DNA databases under a certain discovery condition. An intuitive solution is to use the UO or IMUS algorithm iteratively to perform a complete discovery under all feasible discovery conditions. However, this solution is not sufficiently efficient. The UO and IMUS algorithms are specifically designed for discovering signatures that meet a certain discovery condition, but they cannot discover all of the implicit signatures. Accordingly, an efficient algorithm for discovering all implicit signatures under a certain discovery condition is needed.

The idea of the 'incremental' has been used in many research areas, such as data mining and knowledge discovery [20,21], communications [22-25] and computer graphics and visualization [26,27]. The definitions of the term 'incremental' vary slightly among fields. Here, 'incremental' is used to refer to the fact that a new result is obtained by processing the previously discovered signatures, rather than by performing a complete discovery on the whole database. Additionally, since an increasing number of computers have multi-core processors, parallel computing is applied to accelerate the signature discovery processes. This work proposes an algorithm that is called the Consecutive Multiple Discovery (CMD) algorithm, which is designed specifically for discovering all implicit signatures under a certain discovery condition from DNA databases. The CMD algorithm is an iterative algorithm. It includes an algorithm called Parallel and Incremental Signature Discovery (PISD) algorithm as a kernel routine. The PISD algorithm enhances the hamming-distance-based unique signature discovery algorithms, the UO and IMUS algorithms, by using the incremental and parallel computing techniques. The PISD algorithm is based on observations of hamming-distance-based signatures, and discovers new results by reusing previously discovered signatures but with looser discovery conditions. For example, the algorithm can find signatures of length *l *= 28 and mismatch tolerance *d *= 4 by processing the signatures of *l *= 30 and *d *= 2. The scope of the search is far smaller than the size of the input database. The PISD algorithm runs faster than the typical UO and IMUS algorithms because it reuses the discovered signatures as candidates, rather than all of the patterns in the database. Based on the results from the experiments on human chromosome 13 EST databases, the proposed CMD algorithm discovers all implicit signatures and performs 33.74 times faster than the typical algorithm when eight processing cores are used.

## Results and Discussion

### Algorithm

The proposed Consecutive Multiple Discovery (CMD) algorithm efficiently discovers all of the implicit signatures of length ≤ *l *and mismatch tolerance ≥ *d *under the discovery condition (*l*, *d*). The CMD algorithm uses the parallel and incremental signature discovery (PISD) algorithm as a kernel routine. Given a discovery condition (*l*, *d*), the PISD algorithm is designed for efficiently discovering signatures of length *l' *and tolerance *d'*, and then the CMD algorithm uses the PISD to find all of the implicit signatures of length *l' *≤ *l *and mismatch tolerance *d' *≥ *d*. The PISD algorithm is based on observations of the hamming-distance-based signatures, and uses parallel computing to increase discovery efficiency. The PISD algorithm applies a scheduling heuristic, which is called the parallel entry list (PEL) heuristic, to generate a reordered entry list when parallel computing is used. This entry list improves the performance of the proposed PISD algorithm.

#### The parallel and incremental signature discovery (PISD) algorithm

Let Ω_*l*, *d *_denote the set of the unique signatures discovered by the UO or IMUS algorithm under the discovery condition (*l*, *d*). We have the observations as follows:

**Observation 1. **∀*P *∈ Ω_*l*-1, *d*_, *P *must be a substring of a pattern *Q *in Ω_*l*, *d*_.

Proof.

Assume *P *∈ Ω_*l*-1, *d *_and *P' *is a pattern of length *l *- 1. Since *P *is a signature of condition (*l *- 1, *d*), HD(*P*, *P'*) >*d*, where HD(*P*, *P'*) is the hamming distance between *P *and *P'*.

Let *x *be a character in {A, C, G, T}. Assume *Q *= *x *+ *P *and *Q' *is a pattern of length *l*, where + means string concatenation. HD(*Q*, *Q'*) = HD(*x *+ *P*,  + ) ≥ HD(*P*, ) *> d*, where  is the *i*-th character of *Q' *and  denotes the substring starting from the *i*-th to the *j*-th characters in *Q'*. Hence, *P *is a substring of *Q *and *Q *∈ Ω_*l*, *d *_in this case.

The proof of the case with *Q *= *P *+ *x *can be done in the same way, yielding the result that *P *is a substring of *Q *and *Q *∈ Ω_*l*, *d*_.

Therefore, the observation holds.

**Observation 2. **∀ *P *∈ Ω_*l*, *d*+1_, *P *must be in Ω_*l*, *d*_.

Proof.

Assume *P *∈ Ω_*l*, *d*+1 _and *P' *is a pattern of length *l*. Since *P *∈ Ω_*l*, *d*+1_, HD(*P*, *P'*) >*d *+ 1 >*d*, where HD(*P*, *P'*) is the hamming distance between *P *and *P'*. Thus, *P *∈ Ω_*l*, *d*_. The observation holds.

**Observation 3. **∀*P *∈ Ω_*l*-*a*, *d*+*b*_, *P *must be a substring of a pattern *Q *in Ω_*l*,*d*_, where *a *and *b *are positive integers, and *a *<*l*.

The observations can be used to improve the hamming-distance-based signature discovery algorithms, including the UO and IMUS algorithms. Based on these observations, the unique signatures of factors (*l'*, *d'*) must be discoverable from the unique signatures that satisfy the discovery condition (*l*, *d*), where *l' *≤ *l *and *d' *≥ *d*. Accordingly, the discovery is incremental, reducing the scope of the search in the discovery process. Hereafter, this heuristic is called 'incremental discovery'.

For example, Table 1(A) presents a DNA database of three sequences. Table 1(B) lists the five patterns in the database. Table 1(C) presents Ω_5,1_, Ω_5,2_, Ω_4,1 _and Ω_4,2_. Each pattern in Ω_5,2 _is in Ω_5,1_, and all of the patterns in Ω_4,1 _and Ω_4,2 _are implicit in Ω_5,1_. Restated, to discover Ω_5,2_, Ω_4,1 _or Ω_4,2_, the patterns in Ω_5,1 _can be used as candidates, instead of all of the patterns in the database. Since the number of patterns in Ω_5,1_, 5, is less than the number of patterns in the database, 12, the discovery process is accelerated.

**Table 1:** An example of implicit signatures.

(A) A DNA database.	
CCCTAATG	
TTAATAAT	
ATAATGCG	

**(B) All 5-patterns in the database.**	

CCCTA, CCTAA, CTAAT, TAATG, TTAAT, TAATA	
AATAA, ATAAT, ATAAT, TAATG, AATGC, ATGCG	

**(C) Some unique signatures in the database.**	

Ω_5,1_	CCCTA, CCTAA, AATAA, AATGC, ATGCG

Ω_5,2_	ATGCG

Ω_4,1_	CCCT, CCTA, ATGC, TGCG

Ω_4,2_	TGCG

Let Ω_*l*, *d *_denote the set of the unique signatures of length *l *and mismatch tolerance *d*. The result shows that all of the patterns in Ω_5,2_, Ω_4,1 _and Ω_4,2 _are implicit in Ω_5,1_.

Additional file 1 presents the PISD algorithm. Let *l' *be the desired signature length and *d' *be the mismatch tolerance. Divide all of the DNA sequences in the input database into *α*-patterns, where the value of *α *is related to the selected hamming-distance-based signature discovery algorithm. For example, *α *= *l'*/2 for the IMUS algorithm, and *α *= *l'*/(⌊*d'*/2⌋ + 1) for the UO algorithm. A *l'*-pattern comprises *l'*/*α *consecutive *α*-patterns. An index of 4^*α *^entries is built with the *α*-patterns as index keys. A multi-level index can be adopted if the index is too large to be fit in the main memory. The *l'*-patterns that contain a certain *α*-pattern are collected in an entry. Each entry maintains a list of the locations of the pattern in the database, which is called a pattern list. The patterns in the input database are called data patterns, and the patterns that are discovered by a hamming-distance-based signature discovery algorithm are referred to as candidate patterns. Based on the observations of hamming-distance-based discovery and incremental discovery, the new result obtained under stricter discovery conditions can be discovered from the candidate patterns obtained under looser conditions. To accelerate access, the candidate patterns are arranged in the pattern list in an entry prior to the non-candidate patterns. A pointer indicates the end of the candidate patterns in the pattern list. A processing order list of all of the entries in the index is constructed. If a multiple-processor system is used, then the processing order list is generated by the PEL heuristic (described in the following section); otherwise, the order list includes the entries in an arbitrary order.

**Observation 4. (UO observation) **if two patterns, *P *and *Q*, are (*l'*, *d'*)-mismatched, then at least one of the (⌊*d'*/2⌋ + 1) partitions of *P *is (*α*, 1)-mismatched to the corresponding part in *Q*, where *α *= *l'*/(⌊*d'*/2⌋ + 1) and all partitions have equal length.

**Observation 5. (IMUS observation) **if two patterns *P *and *Q *are (*l'*, *d'*)-mismatched, then at least one of the two halves of *P *is (*α*, ⌊*d'*/2⌋)-mismatched to the corresponding part of *Q*, where *α *= *l'*/2.

Two index entries are called similar entries if the number of mismatches between the keys of the entries is less than or equal to a certain value *β*. This value is also related to the employed discovery algorithm, for example, *β *is 1 in the UO algorithm, and *β *= ⌊*d'*/2⌋ in the IMUS algorithm. Assume *K*_*P *_and *K*_*Q *_are index keys, and *P *and *Q *are the *l'*-patterns listed in the entries of keys *K*_*P *_and *K*_*Q*_, respectively. Based on Observations 4 and 5, if *Q *is (*l'*, *d'*)-mismatched to *P*, then *K*_*Q *_must be (*α*, *β*)-mismatched to *K*_*P*_, such that the entries of keys *K*_*P *_and *K*_*Q *_are similar. Since all the patterns that are (*l'*, *d'*)-mismatched to a pattern *P *must be in the entries that are similar to the entry whose key is *K*_*P*_, *P *is compared to all of the patterns in the similar entries, to determine whether *P *is unique. The pattern *P *is a unique signature if no pattern is (*l'*, *d'*)-mismatched to it. Since the new result can be discovered from the candidate patterns, the PISD processes only the candidate patterns. An available processor is assigned to handle the next untreated entry (based on the assumption that the key of the entry is *K*_*P*_) in the processing order list^. ^Assume that *P *is one of the candidate patterns in the entry. *P *is compared to all of the patterns in the similar entries, which are those whose keys are (*α*, *β*)-mismatched to *K*_*P*_. Each of the comparisons is a complete string comparison of *l' *characters. The candidate *l'*-patterns that are (*l'*, *d'*)-mismatched to any of the *l'*-patterns in the similar entries are discarded, and the remaining candidate patterns are new unique signatures.

#### The scheduling heuristic for parallelism

One of the ways to accelerate signature discovery is to apply parallel computing. Assume that a computer of *n *processors is employed in signature discovery, and that processor *i *takes *t*_*i *_time units to complete its tasks. The overall processing time *T*_*n *_required by the computer to complete the discovery is , which means that the processor that takes longest dominates the overall processing time.

The optimal processing time when *n *processors are used is *T*_*n *_= *T*_1_/*n*, which equals 1/*n *of the processing time of a single-processor computer.

The simplest way to apply parallel computing to the proposed PISD algorithm is to assign randomly an available processor to process the patterns in the index in an arbitrary order. The treatment of an entry is referred to as a task. For example, a computer with four processors is used to handle *N *tasks. Processor 1 can be assigned to task 1, ..., and processor 4 can be assigned to task 4. Assume that processor 3 is the first to complete its task; the processor is immediately assigned to the next task, task 5. The next available processor is similarly assigned to the next task until all of the *N *tasks are completed. If four tasks are processed simultaneously, then ideally, the overall processing time is reduced to one quarter of that which would be required using a single-processor computer.

**Figure 1:**
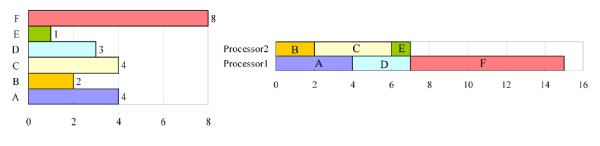
**An example of the first potential problem of parallel signature discovery**. The tasks can be completed in 22 time units by a single-processor computer. The overall processing time is 15 units for a two-processor computer, which exceeds the optimal processing time, 11 units.

**Figure 2:**
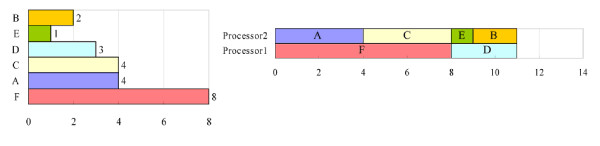
**The result of moving long tasks forward in the processing order list**. The long tasks are moved forward in the processing order list in Figure 2, yielding the new processing order list. The overall processing time for a two-processor computer is 11 units, which equals the optimal processing time.

However, two potential problems must be considered when parallel computing is applied to the proposed PISD algorithm. First, if one of the last few tasks requires much processing time, then the overall processing time may be longer than the optimal processing time. For example, Figure 1 shows a list of six tasks. All of the tasks can be completed in 22 time units by a single-processor computer. The optimal processing time is therefore 22/2 = 11 units for a two-processor computer. However, in this case, processor 1 is assigned to {A, D, F}, and processor 2 is assigned to {B, C, E}. The processing times are 15 and 7 units respectively, and the overall processing time is 15 units, which exceeds the optimal processing time. This situation can be avoided by arranging long tasks before the others in the processing order list. Here, the long tasks are moved forward in the processing order list, yielding the result in Figure 2. In the new list, processor 1 performs tasks {F, D} and processor 2 performs tasks {A, C, E, B}. The overall processing time is 11 units, which equals the optimal processing time.

**Figure 3:**
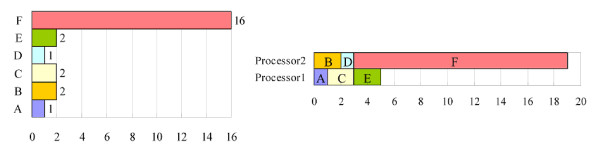
**An example of the second potential problem of parallel signature discovery**. The tasks can be completed in 24 units by a single-processor computer. The overall processing time is 19 units for a two-processor computer, which exceeds the optimal processing time, 12 units.

**Figure 4:**
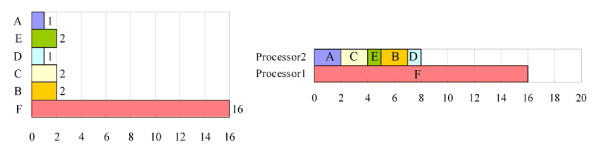
**The result of moving long tasks forward in the processing order list**. The long tasks are moved forward in the processing order list in Figure 4, yielding the new processing order list. The overall processing time for a two-processor computer is 16 units, which still exceeds the optimal processing time.

**Figure 5:**
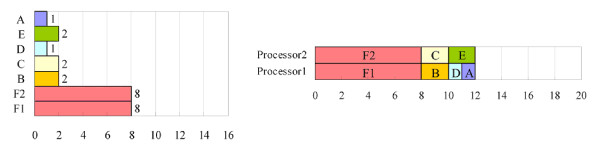
**The result of dividing long tasks into short tasks in the processing order list**. Task F in Figure 5 is divided into two tasks with identical processing times, yielding the new task list. The overall processing time for a two-processor computer is 12 units, which equals the optimal processing time.

The second potential problem is that the time required to process a task may exceed the optimal processing time, *T*_1_/*n*. For example, Figure 3 shows a list of six tasks. All of the tasks can be completed in 24 units by a single-processor computer. When a two-processor computer is used to handle the tasks, processors 1 and 2 are assigned to tasks {A, C, E} and {B, D, F}, and taking 5 and 19 units, respectively. The overall processing time is 19 units. Long tasks are moved forward, yielding the new processing order list that is shown in Figure 4. In this situation, processor 1 is assigned to task F only, and processor 2 is assigned to the other tasks. The overall processing time is then 16 units, which still exceeds the optimal processing time, because task F takes 16 units, which exceeds the sum of the times required to complete all of the other tasks. Hence if less time were to be spent on task F, then the overall processing time would be reduced. Generally, when an entry has more patterns than the other entries, a task that handles this entry takes more time to complete. Therefore, some of the longest entries are divided into *n *equal partitions, which are then treated as typical entries, where *n *is the number of available processors. For example, task F in Figure 4 can be divided into two tasks with identical processing times, yielding the new task list in Figure 5. After the division, processor 1 is assigned to tasks {F_1_, B, D, A}, and processor 2 is assigned to tasks {F_2_, C, E}. The overall processing time is 12 units, which equals the optimal processing time.

Based on the above discussion, the order of tasks in the processing order list influences the overall processing time for parallel discovery. Since the proposed discovery algorithm PISD focuses on processing candidate patterns, the processing time of a task is proportional to the number of candidate patterns in the entry. The index entries can be sorted in descending order of the number of candidate patterns therein, and the sorted list can be used as the processing order list. Entries that contain more candidate patterns are expected to be at the top of the list. However, the sorting process takes *O*(*N *log *N*) time for *N *entries, which is significant.

A simple and efficient scheduling heuristic, called the parallel entry list (PEL), is provided. It yields a processing order list for tasks in which the tasks that involve more candidate patterns are before those that involve fewer. Additional file 2 displays the PEL heuristic. The PEL heuristic is similar to a partial quicksort. Unlike quicksort, the PEL heuristic is iterative, and only operates on the left part of a list in each iteration. Firstly, the PEL heuristic generates a processing order list *L *that consists of all of the index entries in arbitrary order, and *w *is defined as the number of index entries in *L*. The average number of candidate patterns (*g*) in each entry is computed, where *g *equals (total number of candidate patterns)/*w*. Let *L*_*i *_represent the *i*-th entry in *L*, and || be the number of candidate patterns in *Li*. Then, the PEL heuristic searches for the maximal value *r *such that || >*g *and the minimal value *k *such that || ≤ *g*, and then exchanges *L*_*k *_and *L*_*r*_. The searches and exchanges continue until *r *<*k*. The process scans the entries from *L*_1 _to *L*_*w *_in *L *and *w *is updated to the current value of *r*. Then, the entries in *L *are divided into two parts: if *i *≤ *w*, then || >*g*; otherwise, || ≤ *g*. Assume *w' *is the most recent value of the variable *w*. Since || >*g*, ∀ *i *≤ *w*, *w *<*w'*/2. Then, the PEL heuristic focuses on the first part of *L*, and moves the long entries forward until , where *n *is the number of available processors and *N *is the number of index entries in *L*. Now, the first *w *entries in *L *are the top *w *entries, which contain the most candidate patterns. The first *w *entries are removed from *L*, and the candidate patterns in each entry are divided into *n *partitions of equal number of patterns. The *nw *partitions are then put into *L*, and treated as typical entries in successive processes. The total number of scans performed on is , where *m *is the number of iterations of the main outer loop (line 10 to 27 of the PEL heuristic in Additional file 2), which moves the long entries forward. The time complexity of the PEL heuristic is *O*(3*N*) = *O*(*N*).

As an example of the above, consider an entry list *L*, shown in Table 2(A). The average number of candidate patterns in each entry (*g*) is 41. The leftmost entry in *L *that contains fewer than *g *candidate patterns, and the rightmost entry that contains more than *g *candidate patterns are sought. The respective results are entries A and J. These two entries are exchanged in *L*. Entries B and G as well as D and E are similarly exchanged. Table 2(B) shows the new processing order list. Now, *w *is four, and the number of candidate patterns in each of the first *w *= 4 entries exceeds *g *= 41, while that in the other entries is less than 41. Then, only the region of the first four entries is considered in the next step. The average number of the candidate patterns in each entry within this region is computed, yielding *g *= 79. In this region, the leftmost and rightmost entries that contain fewer than and more than 79 candidate patterns are J and E, respectively. J and E are exchanged in the list, yielding Table 2(C). Assume a two-processor computer is used. Entry E is divided into two partitions E_1 _and E_2_, and E_1 _and E_2 _are added to the list. The new list is as shown in Table 2(D). Processor 1 will handle entries E_1_, G, D, F, B, I and A, and processor 2 will handle entries E_2_, C, J, H and K. The total number of candidate patterns to be treated by each processor is 227.

**Table 2:** An example of using the PEL heuristic to build an entry list.

(A) The original entry list.												
**ID**	**A**	**B**	**C**	**D**	**E**	**F**	**G**	**H**	**I**	**J**	**K**	

|*|	33	26	49	5	143	9	72	29	11	55	22	

**(B) After first iteration, *w *= 4.**												

**ID**	**J**	**G**	**C**	**E**	**D**	**F**	**B**	**H**	**I**	**A**	**K**	

|*|	55	72	49	143	5	9	26	29	11	33	22	

**(C) After second iteration, *w *= 1.**												

**ID**	**E**	**G**	**C**	**J**	**D**	**F**	**B**	**H**	**I**	**A**	**K**	

|*|	143	72	49	55	5	9	26	29	11	33	22	

**(D) The final entry list.**												

**ID**	**E_1_**	**E_2_**	**G**	**C**	**J**	**D**	**F**	**B**	**H**	**I**	**A**	**K**

|*|	71	72	72	49	55	5	9	26	29	11	33	22

Let |*| denote the number of candidate patterns in an entry. Part (A) presents the original entry list. Entries A and J, B and G as well as D and E are exchanged. Part (B) presents the new entry list. Entries J and E are exchanged in the next iteration, yielding Part (C). Assume a two-processor computer is used. Entry E is divided into two partitions E_1 _and E_2_, and E_1 _and E_2 _are added to the list. The new list is as shown in Part (D).

#### The consecutive multiple discovery (CMD) algorithm

Additional file 3 displays the consecutive multiple discovery (CMD) algorithm. Let *l *and *d *be two integers. The CMD algorithm is an iterative algorithm, which uses the PISD algorithm as a kernel routine, to discover all implicit signatures under the discovery condition of length *l *and mismatch tolerance *d*. Firstly, the UO or IMUS algorithm is used to discover the unique signatures that satisfy the discovery condition (*l*, *d*). The signatures discovered by UO or IMUS are applied as candidates in successive discoveries. The feasible discovery conditions are all combinations of the possible *l' *and *d'*, which means {(*l' *≤ *l*, *d' *≥ *d*)}. In each discovery, the PISD algorithm is used to discover new signatures from the candidates under a feasible discovery condition. The discovery process continues until all of the implicit signatures are discovered.

### Testing

This section evaluates the performance of the proposed algorithms. Since the incremental discovery and parallel computing mentioned in the previous sections can be applied to the UO and IMUS algorithms, briefly, the CMD (or PISD) with the UO and IMUS kernels are denoted as CMD_UO _and CMD_IMUS _(or PISD_UO _and PISD_IMUS_), respectively. The algorithms are analyzed based on a uniformly distributed database. The first part of this section presents these analyses. To evaluate the performance of the UO, IMUS, CMD_UO _and CMD_IMUS _algorithms, they are applied to human chromosome 13 and 21 EST databases for signature discovery. The second part of this section presents the experimental results.

#### Mathematical analyses

The CMD algorithm is an iterative algorithm. It includes the PISD algorithm as a kernel routine. Accordingly, the time complexity of the PISD algorithm dominates that of the CMD algorithm. First, the time complexity of the PISD_UO _algorithm is analyzed under a certain discovery condition, and then, the results are integrated, yielding the time complexity of the CMD_UO _algorithm. The analyses of the PISD_IMUS _and CMD_IMUS _algorithms can be done in a similar way.

Let *l' *be the signature length and *d' *be the mismatch tolerance. *σ*_*l' *_denotes the index system built under the condition of signature length *l' *in the PISD_UO _algorithm. *σ*_*l' *_consists of 4^*α *^pattern entries, where *α *= *l'*/(⌊*d'*/2⌋ + 1) is the length of the entry keys. Let *σ*_*l'*, *i *_be the *i*-th entry in *σ*_*l'*_·|*σ*_*l'*, *i*_| denotes the number of all patterns in *σ*_*l'*, *i *_and  denotes the number of candidate patterns in *σ*_*l'*, *i*_. HD(*σ*_*l'*, *i*_, *σ*_*l'*, *j*_) denotes the hamming distance between *σ*_*l'*, *i *_and *σ*_*l'*, *j*_, which is defined as the hamming distance between the entry keys of *σ*_*l'*, *i *_and *σ*_*l'*, *j*_. Because only the candidate patterns have to be considered, |*σ*_*l'*, *i*_| string comparisons are performed on the patterns in *σ*_*l'*, *i*_. Additionally, ∑_*j*_|*σ*_*l'*, *j*_| string comparisons are required to check possible mutants, where *σ*_*l'*, *j *_∈ *σ*_*l' *_such that HD(*σ*_*l'*, *i*_, *σ*_*l'*, *j*_) = 1. All characters in an *l'*-pattern excluding the entry key region are compared in each of the string comparisons, yielding *l' *- *α *character comparisons.

The total amount of character comparisons used in the PISD_UO _algorithm, denoted as , is:

where *σ*_*l'*, *j *_∈ *σ*_*l' *_such that HD(*σ*_*l'*, *i*_, *σ*_*l'*, *j*_) = 1.

Let *l *be the desired signature length and *d *be the mismatch tolerance of pattern uniqueness. the CMD_UO _algorithm uses the PISD_UO _algorithm to find all of the implicit signatures of length *l' *≤ *l *and mismatch tolerance *d' *≥ *d*. The time complexity of the CMDUO algorithm, denoted as , is:

where *σ*_*l'*, *j *_∈ *σ*_*l' *_such that HD(*σ*_*l'*, *i*_, *σ*_*l'*, *j*_) = 1.

Assume the input DNA database *D *and the set of the discovered signatures Ω_*l' *≤ *l*, *d' *≥ *d *_are uniformly distributed. Let  ∈ {Ω_*x*, *y*_}*l' *≤ *x *≥ *l *and *d *≤ *y *≥ *d'*}, where (*x*, *y*) ≠ (*l'*, *d'*), be the set of signatures discovered in the strictest iteration prior to the iteration of (*l'*, *d'*). Assume the sizes of *D *and  are denoted as |*D*| and ||. The index system built in this uniformly distributed case is denoted as . Each entry in  should contain || ≈ |*D*|/4^*α *^patterns, and  of them are candidate patterns. In this case, the amount of character comparisons used in the PISD_UO _algorithm, denoted as , is:

where *σ*_*l'*, *j *_∈ *σ*_*l' *_such that HD(σ_*l'*, *i*_, *σ*_*l'*, *j*_) = 1, and *κ *= 3*α *is the number of all possible 1 base permutations of a string of length *α*. Note that  because of the uniform assumption.

In the uniformly distributed case, the number of character comparisons used in the CMD_UO _algorithm, denoted as , is:

where *κ *= 3*α*.

The main difference between the PISD_UO _and the typical UO algorithms is that the two algorithms use different candidate sets. The PISD_UO _algorithm uses the previously discovered signatures as candidates, but the UO algorithm uses all of the patterns in the database as candidates. The amount of character comparisons used in the UO algorithm for discovering signatures from the uniformly distributed database *D *can be obtained by replacing  with *D*. The formula is:

where *κ *= 3*α*.

The UO algorithm is executed repeatedly to discover all implicit signatures. The time complexity of using the UO algorithm, denoted as , is:

where *κ *= 3*α*.

The gain delivered by the CMD_UO _algorithm is:

where *κ *= 3*α*.

It means that the CMD_UO _algorithm performs *G *≥ |*D*|/|Ω_*l*, *d*_| times faster than the typical UO algorithm, when discovering implicit signatures from a uniformly distributed database.

#### Performance evaluation

The platform that was adopted in this experiment was a Dell PowerEdge R900 server with two Intel Xeon E7430 2.13 GHz quad-core CPUs, 12 GB RAM and 900 GB disk space. The operating system was Red Hat Enterprise Linux 5. The algorithms were implemented in JAVA language, and the programs were compiled by JDK 1.6. The DNA data that were used in the experiments were from the human chromosome 13 and 21 EST databases. Before the experiments, the remarks in the databases were removed; all of the universal characters, such as 'don't care', were replaced with 'A', and DNA sequences that were shorter than 36 bases were discarded. The experimental data are denoted as *D*_13 _(human chromosome 13 EST database) and *D*_21 _(human chromosome 21 EST database), and their corresponding sizes were approximately 36.44 M and 22.21 M bases.

The pooled oligo probes, that are used to screen an EST library, such as the BAC library, generally have lengths from 24 to 40 bases [28]. Our experimental results on unique signature discoveries, with the criteria of exact matches, also shows that most of the human EST sequences can be distinctly labeled by signatures of length greater than 18 bases. Accordingly, the experiments in this section focused on discovering signatures of length between 24 and 30 with mismatch tolerances of two and four.

For reasons of performance and memory consumption, a two-level index was used in the implementation of the IMUS and CMD_IMUS _algorithms. The first level of the index comprised 4^10 ^direct-accessible entries, and a binary search was used to locate a specified entry in the second level. The index systems that were used in the implementation of the UO and CMD_UO _algorithms were one-level, and all of the entries in their index systems were directly accessible. Since the purpose of our experiments was to evaluate the improvements provided by incremental discovery and parallel computing, additional filters, such as the frequency filter that was used in the IMUS algorithm, was excluded from the kernels of the algorithms.

Since ⌊*d*/2⌋ + 1 = 2 when *d *= 2, the kernels of the UO and IMUS algorithms are very similar under this condition. Only the performance of the IMUS and CMD_IMUS _algorithms was examined when mismatch tolerance was two. Table 3 presents the discovery conditions that were used in our experiments. In the experiments on the UO and IMUS algorithms, the UO and IMUS algorithms were executed repeatedly to discover all of the signatures under all feasible discovery conditions. The experiments were performed on a one-processor computer. Before the performance of the CMD_UO _and CMD_IMUS _algorithms was evaluated, the IMUS algorithm was used to discover signatures under the discovery condition of length *l *= 30 and mismatch tolerance *d *= 2. The discovery on *D*_13 _took approximately 19.6 minutes and 20.88% of the patterns from *D*_13 _were discovered as signatures. The discovery on *D*_21 _took about 5.3 minutes and 22.07% of the patterns from *D*_21 _were discovered as signatures. In each successive experiment, the CMD_UO _and CMD_IMUS _algorithms used the discovered signatures of *l *= 30 and *d *= 2 as candidates to produce new results.

**Table 3:** The discovery conditions used in our experiments.

The used discovery conditions.								
**(*l'*, *d'*)**	**(28,2)**	**(26,2)**	**(24,2)**	**(30,4)**	**(28,4)**	**(27,4)**	**(26,4)**	**(24,4)**

CMD_UO_				•		•		•

CMD_IMUS_	•	•	•	•	•	•	•	•

• indicates that the discovery condition was used in the experiments by the specified algorithm.

The percentage time saved is used to evaluate the improvements in the processing time of an algorithm. The time saving is defined as (1-(processing time of the CMD_UO _(or CMD_IMUS_) algorithm)/(processing time of the UO (or IMUS) algorithm))*100%. A larger 'saving' means a greater improvement by the CMD_UO _or CMD_IMUS _algorithm. The term 'overall' refers to the total processing time required for the UO, IMUS, CMD_UO _or CMD_IMUS _algorithm to discover all of the signatures that satisfy the discovery conditions.

First, improvements in the time of discovery associated with incremental discovery are examined. For a single processing core, the performance of the CMD_UO _and CMD_IMUS _algorithms was evaluated by using the algorithms to discover signatures from *D*_13 _and *D*_21_. Tables 4 and 5 present the processing time that for the UO and IMUS algorithms, and the time savings delivered by the CMD_UO _and CMD_IMUS _algorithms. The tables also present the processing time required to discover signatures under every discovery condition. In the experiments, the proposed CMD_UO _algorithm took 76.2% less processing time than the UO algorithm to discover all of the implicit signatures from *D*_13_, and about 74% less processing time to discover those from *D*_21_. With respect to the performance of the CMD_IMUS _algorithm, it took about 67% and 52% less processing time than the IMUS algorithm to discover all of the signatures from *D*_13 _and *D*_21_. Greater overheads in accessing indices caused the percentage processing time saved by the CMD_IMUS _algorithm to be less than that saved by the CMD_UO _algorithm.

**Table 4:** The performance of the CMD_UO _algorithm when using a single processing core.

(A) The results on *D*_13_.				
**(*l'*, *d'*)**	**(30,4)**	**(27,4)**	**(24,4)**	**overall**

UO	03:49:54	09:56:59	35:56:27	49:43:20

CMD_UO_	00:50:52	02:22:32	08:37:03	11:50:27

saving(%)	77.87	76.12	76.02	76.19

**(B) The results on *D*_21_.**				

**(*l'*, *d'*)**	**(30,4)**	**(27,4)**	**(24,4)**	**overall**

UO	01:10:18	03:09:37	11:12:18	15:32:13

CMD_UO_	00:17:10	00:49:34	02:55:27	04:02:11

saving(%)	75.58	73.86	73.90	74.02

The table presents the processing time that for the UO algorithm, and the time savings delivered by the CMD_UO _algorithm. The time format used in the table is HH:MM:SS.

**Table 5:** The performance of the CMD_IMUS _algorithm when using a single processing core.

(A) The results on *D*_13_.								
**(*l'*, *d'*)**	**(28,2)**	**(26,2)**	**(24,2)**	**(30,4)**	**(28,4)**	**(26,4)**	**(24,4)**	**overall**

IMUS	00:23:56	00:30:24	00:43:35	00:50:57	01:10:14	01:57:30	03:57:25	09:34:01

CMD_IMUS_	00:03:06	00:04:54	00:08:33	00:20:22	00:27:09	00:44:03	01:21:22	03:09:29

saving(%)	87.03	83.84	80.35	60.01	61.33	62.50	65.73	66.99

**(B) The results on *D*_21_.**								

**(*l'*, *d'*)**	**(28,2)**	**(26,2)**	**(24,2)**	**(30,4)**	**(28,4)**	**(26,4)**	**(24,4)**	**overall**

IMUS	00:06:25	00:08:22	00:11:48	00:19:17	00:25:08	00:40:13	01:16:54	03:08:07

CMD_IMUS_	00:01:27	00:02:13	00:03:48	00:11:08	00:13:38	00:20:17	00:37:28	01:29:59

saving(%)	77.43	73.61	67.81	42.24	45.80	49.56	51.28	52.17

The table presents the processing time that for the IMUS algorithm, and the time savings delivered by the CMD_IMUS _algorithm. The time format used in the table is HH:MM:SS.

To elucidate the benefits of parallel computing for signature discovery, various number of processing cores were used and the PISD_UO _and PISD_IMUS _algorithms were used to discover the signatures of (*l' *= 24, *d' *= 4) from *D*_13_. Table 6 shows the experimental results: the acceleration is the processing time normalized to the processing time when one processor is used. When the PISD_UO _algorithm is used, the acceleration of the discovery processes is almost proportional to the number of processing cores used. The acceleration values of the PISD_IMUS _algorithm increase with the number of processing cores such that the discovery process using eight processing cores is approximately 4.6 times faster than that using a single core.

**Table 6:** The benefits of parallel computing for signature discovery.

(A) PISD_UO_.				
**CPUs**	**1**	**2**	**4**	**8**

Time	08:37:03	04:22:04	02:07:22	01:04:23

Acceleration	1.00	1.97	4.06	8.03

**(B) PISD_IMUS_.**				

**CPUs**	**1**	**2**	**4**	**8**

Time	01:21:22	00:52:24	00:28:33	00:17:36

Acceleration	1.00	1.55	2.85	4.62

With various number of processing cores, the PISD_UO _and PISD_IMUS _algorithms were used to discover the signatures of (*l' *= 24, *d' *= 4) from *D*_13_. The table shows the experimental results. The acceleration is the processing time normalized to the processing time when one processor is used. The time format used in the table is HH:MM:SS.

Finally, the improvements in the discovery performance delivered by a combination of incremental discovery and parallel computing are examined. In this case, the CMD_UO _and CMD_IMUS _algorithms discovered signatures from the databases using eight processing cores. Tables 7 and 8 present the time savings made by the CMD_UO _and CMD_IMUS _algorithms. Tables 9 and 10 show the number of discovered signatures under each discovery condition. In the experiments, the proposed CMD_UO _algorithm took 97% less processing time than the UO algorithm to discover all of the implicit signatures from *D*_13_, and about 96.7% less processing time to complete discovery on *D*_21_. The CMD_IMUS _algorithm took about 92.6% and 88.8% less processing time than the IMUS algorithm, to discover all of the signatures from the experimental data *D*_13 _and *D*_21_, respectively.

**Table 7:** The performance of the CMD_UO _algorithm when using eight processing cores.

(A) The results on *D*_13_.				
**(*l'*, *d'*)**	**(30,4)**	**(27,4)**	**(24,4)**	**overall**

UO	03:49:54	09:56:59	35:56:27	49:43:20

CMD_UO_	00:06:27	00:17:36	01:04:23	01:28:26

saving(%)	97.19	97.05	97.01	97.04

**(B) The results on *D*_21_.**				

**(*l'*, *d'*)**	**(30,4)**	**(27,4)**	**(24,4)**	**overall**

UO	01:10:18	03:09:37	11:12:18	15:32:13

CMD_UO_	00:02:13	00:06:03	00:22:20	00:30:36

saving(%)	96.85	96.81	96.68	96.72

The CMD_UO _algorithm discovered signatures from the databases using eight processing cores. The table presents the time savings made by the CMD_UO _algorithm. The time format used in the table is HH:MM:SS.

**Table 8:** The performance of the CMD_IMUS _algorithm when using eight processing cores.

(A) The results on *D*_13_.								
**(*l'*, *d'*)**	**(28,2)**	**(26,2)**	**(24,2)**	**(30,4)**	**(28,4)**	**(26,4)**	**(24,4)**	**overall**

IMUS	00:23:56	00:30:24	00:43:35	00:50:57	01:10:14	01:57:30	03:57:25	09:34:01

CMD_IMUS_	00:00:40	00:00:59	00:01:33	00:04:58	00:06:31	00:10:18	00:17:36	00:42:35

saving(%)	97.21	96.77	96.44	90.25	90.72	91.23	92.59	92.58

**(B) The results on *D*_21_.**								

**(*l'*, *d'*)**	**(28,2)**	**(26,2)**	**(24,2)**	**(30,4)**	**(28,4)**	**(26,4)**	**(24,4)**	**overall**

IMUS	00:06:25	00:08:22	00:11:48	00:19:17	00:25:08	00:40:13	01:16:54	03:08:07

CMD_IMUS_	00:00:20	00:00:30	00:00:43	00:02:44	00:03:22	00:04:57	00:08:29	00:21:05

saving(%)	94.81	94.02	93.93	85.83	86.60	87.69	88.97	88.79

The CMD_IMUS _algorithm discovered signatures from the databases using eight processing cores. The table presents the time savings made by the CMD_IMUS _algorithm. The time format used in the table is HH:MM:SS.

**Table 9:** The number of signatures discovered by the CMD_UO _algorithm when using eight processing cores.

(A) The number of discovered signatures in *D*_13_.			
**(*l'*, *d'*)**	**(30,4)**	**(27,4)**	**(24,4)**

UO	4018054	3918976	3401911

CMD_UO_	4018054	3918976	3401911

**(B) The number of discovered signatures in *D*_21_.**			

**(*l'*, *d'*)**	**(30,4)**	**(27,4)**	**(24,4)**

UO	2581787	2525108	2277644

CMD_UO_	2581787	2525108	2277644

The CMD_UO _algorithm discovered signatures from the databases using eight processing cores. The table presents the number of discovered signatures under each discovery condition.

**Table 10:** The number of signatures discovered by the CMD_IMUS _algorithm when using eight processing cores.

(A) The number of discovered signatures in *D*_13_.							
**(*l'*,*d'*)**	**(28,2)**	**(26,2)**	**(24,2)**	**(30,4)**	**(28,4)**	**(26,4)**	**(24,4)**

IMUS	4676722	4661305	4612508	4018054	3967920	3836732	3401911

CMD_IMUS_	4676722	4661305	4612508	4018054	3967920	3836732	3401911

**(B) The number of discovered signatures in *D*_21_.**							

**(*l'*,*d'*)**	**(28,2)**	**(26,2)**	**(24,2)**	**(30,4)**	**(28,4)**	**(26,4)**	**(24,4)**

IMUS	3017278	3010587	2982960	2581787	2552522	2482618	2277644

CMD_IMUS_	3017278	3010587	2982960	2581787	2552522	2482618	2277644

The CMD_IMUS _algorithm discovered signatures from the databases using eight processing cores. The table presents the number of discovered signatures under each discovery condition.

The experimental results reveal that the CMD_UO _and CMD_IMUS _algorithms with one processing core require up to 76% and 67% less processing time to find all implicit signatures than the typical UO and IMUS algorithms, respectively. Moreover, up to 97% and 93% of the processing time is saved when the CMD_UO _and CMD_IMUS _algorithms are executed using eight processing cores. Restated, the proposed CMD_UO _and CMD_IMUS _algorithms perform 4.2 and 3.03 times faster than the typical UO and IMUS algorithms when one processing core is used, and 33.74 and 13.48 times faster when eight processing cores are used.

## Conclusions

This work proposes two unique signature discovery algorithms - the consecutive multiple discovery (CMD) algorithm and the parallel and incremental signature discovery (PISD) algorithm. The CMD algorithm is designed to discover all implicit signatures from DNA databases, providing all implicit signatures to users, especially when they are using an unfamiliar DNA database. The PISD algorithm is a parallel and incremental enhancement of existing signature discovery algorithms. It is based on incremental discovery, and efficiently discovers signatures under a certain discovery condition. This incremental strategy can be adapted to all hamming-distance-based unique signature discovery algorithms. The PISD algorithm has a significantly shorter processing time for signature discovery than typical discovery algorithms. The PISD algorithm is the kernel of the CMD algorithm. Consequently, the CMD algorithm provides an efficient means of implicit signature discovery.

## Authors' contributions

HPL carried out the unique signature studies, participated in the design of the study, programmed the algorithms, evaluated the experimental results and drafted the manuscript. TFS participated in its design and coordination, performed the mathematical analysis and drafted the manuscript. CYT convinced of the study and helped to gather data. All authors read and approved the final manuscript.

## Supplementary Material

Additional file 1 **Parallel and Incremental Signature Discovery (PISD) algorithm. **Assume *l' *is the desired signature length and *d' *is the mismatch tolerance. *α *and *β *are two integers that are related to the selected hamming-distance-based signature discovery algorithm. *α *= *l'*/2 and *β *= ⌊*d'*/2⌋ for the IMUS algorithm, and *α *= *l'*/(⌊*d'*/2⌋ + 1) and *β *= 1 for the UO algorithm. The algorithm is designed for efficiently discovering signatures under the discovery condition (*l'*, *d'*). Click here for file

Additional file 2 **Parallel Entry List (PEL) heuristic. **The heuristic yields a processing order list for index entries in which the entries that involve more candidate patterns are before those that involve fewer. The reordered entry list improves the performance of the proposed PISD algorithm. Click here for file

Additional file 3 **Consecutive Multiple Discovery (CMD) algorithm. **Let *l *be the desired signature length and *d *be the mismatch tolerance of pattern uniqueness. The algorithm discovers all implicit signatures under the discovery condition (*l*, *d*). Click here for file
