# Supplementary material for: A parallel and incremental algorithm for efficient unique signature discovery on DNA databases
Source: BMC Bioinformatics. 2010 Mar 16;11:132. doi: 10.1186/1471-2105-11-132 (PMC2848650; doi:10.1186/1471-2105-11-132)
Supplement: Additional file 1 — Parallel and Incremental Signature Discovery (PISD) algorithm. Assume l' is the desired signature length and d' is the mismatch tolerance. α and β are two integers that are related to the selected hamming-distance-based signature discovery algorithm. α = l'/2 and β = ⌊d'/2⌋ for the IMUS algorithm, and α = l'/(⌊d'/2⌋ + 1) and β = 1 for the UO algorithm. The algorithm is designed for efficiently discovering signatures under the discovery condition (l', d'). [file 1471-2105-11-132-S1.PDF]

```
S ← divide all of the DNA sequences in the input database into α-patterns
σ_l' ← construct an index of 4^α entries with the α-patterns in S as index keys
if the number of available processors > 1 then
  L ← generate a processing order list of all of the entries in σ_l' by the PEL
  heuristic
else
  L ← construct a processing order list of all of the entries in σ_l' in an
  arbitrary order
end if
for an entry E in L do
  assign an available processor to handle E
  K_P ← the entry key of E
  for a candidate pattern P in E do
    for an entry E' whose key is (α, β)-mismatched to K_P do
      compare P to all of the patterns in E'
      if P is (l', d')-mismatched to any of the compared patterns then
        discard P
      end if
    end for
  end for
end for
mark the remaining candidate patterns as the signatures of (l', d')
```
