# Supplementary material for: A parallel and incremental algorithm for efficient unique signature discovery on DNA databases
Source: BMC Bioinformatics. 2010 Mar 16;11:132. doi: 10.1186/1471-2105-11-132 (PMC2848650; doi:10.1186/1471-2105-11-132)
Supplement: Additional file 2 — Parallel Entry List (PEL) heuristic. The heuristic yields a processing order list for index entries in which the entries that involve more candidate patterns are before those that involve fewer. The reordered entry list improves the performance of the proposed PISD algorithm. [file 1471-2105-11-132-S2.PDF]

```
L ← generates a processing order list that consists of all of the index entries
in arbitrary order
n ← the number of available processors
w ← the number of entries in L
s←0, r←w
for i ← 1 to w do
  s ← s + |L*_i|
end for
g ← s/w
h ← g
while g < nh do
  s←0, k←1
  while r > k do
    while |L*_r| ≤ g do
      r ← r − 1
    end while
    while |L*_k| > g do
      s ← s + |L*_k|
      k ← k + 1
    end while
    if r > k then
      exchange L_k and L_r
      s ← s + |L*_k|
    end if
  end while
  w ← r
  g ← s/w
end while
for i ← 1 to w do
  Y ← L_i
  divide Y into n partitions Y_1, Y_2, ..., Y_n
  remove L_i from L
  put Y_1, Y_2, ..., Y_n into L
end for
return L
```
