# Supplementary material for: A parallel and incremental algorithm for efficient unique signature discovery on DNA databases
Source: BMC Bioinformatics. 2010 Mar 16;11:132. doi: 10.1186/1471-2105-11-132 (PMC2848650; doi:10.1186/1471-2105-11-132)
Supplement: Additional file 3 — Consecutive Multiple Discovery (CMD) algorithm. Let l be the desired signature length and d be the mismatch tolerance of pattern uniqueness. The algorithm discovers all implicit signatures under the discovery condition (l, d). [file 1471-2105-11-132-S3.PDF]

$\Omega_{l,d} \leftarrow$ the signatures discovered by the UO or IMUS algorithm under the condition (l, d)

for each feasible discovery condition (l', d'), where $l' \leq l$ and $d' \geq d$ **do**

use the PISD algorithm to discover signatures from $\Omega_{l,d}$ under the condition (l', d')

end for
